# Designed Fabrication of Phloretin-Loaded Propylene Glycol Binary Ethosomes: Stability, Skin Permeability and Antioxidant Activity

**DOI:** 10.3390/molecules29010066

**Published:** 2023-12-21

**Authors:** Meng Zhang, Xue Zhuang, Siqi Li, Yansong Wang, Xiangyu Zhang, Jinlian Li, Dongmei Wu

**Affiliations:** 1 College of Pharmacy, Jiamusi University, Jiamusi 154007, China; 13394549572@163.com (M.Z.); 15765345726@163.com (X.Z.); 15344549861@163.com (S.L.); 16645440927@163.com (Y.W.); lijinlian@jmsu.edu.cn (J.L.); 2 Heilongjiang Provincial Key Laboratory of New Drug Development and Pharmacotoxicological Evaluation, Jiamusi University, Jiamusi 154007, China

**Keywords:** binary ethosomes, propylene glycol, skin permeability, antioxidant activity

## Abstract

Binary ethosome vesicles have been developed as flexible lipid vesicles for the enhanced physicochemical stability and skin delivery of drugs. This work aimed to prepare phloretin-loaded propylene glycol ethosomes (PHL-PGEs) to improve their stability, skin permeability and antioxidant activity. PHL-PGEs were prepared via the ethanol injection method and optimized using different weight ratios of ethanol to propylene glycol (PG). When the ethanol/PG mass ratio changed from 10:0 to 0:10, the encapsulation efficiency and stability of ethosomes increased. At a PHL concentration of 1mg/mL, the EE% was 89.42 ± 2.42 and the DL% was 4.21 ± 0.04, which exhibited their highest values. The encapsulation of the PHL in the PHL-PGEs was strengthened via XRD analysis and FTIR analysis. The results of the in vitro percutaneous permeability test demonstrated that the combined use of ethanol and PG exhibited a notable enhancement in skin permeability, and the skin retention of PHL-PGEs was 1.06 times that of PHL-ethosomes (PHL-Es) and 2.24 times that of the PHL solution. An in vitro antioxidant activity study indicated that solubility and antioxidant activity was potentiated via the nanoencapsulation of phloretin. Therefore, these results confirm the potential of this nanocarrier to enhance physicochemical stability, skin permeability and antioxidant activity.

## 1. Introduction

The formulation of new devices to be used as transdermal drug delivery systems with exceptional stability and permeability is an important field in biomedicine. Ethosomes are an innovative type of liposome that are composed of phospholipids, water and a high proportion of ethanol (EtOH). Ethanol and lipid molecules act in the polar head group region, which can force the stratum corneum lipids on the skin to alter their molecules, increase lipid fluidity, enhance the flexibility and permeability of the ethosomes membranes, and significantly improve skin delivery [1,2,3,4]. Ethosomes can carry hydrophilic substances in their internal aqueous phase and hydrophobic substances within a double membrane, with a negative charge due to ethanol that leads to a reduction in vesicle particle size, and that increases the bioavailability of the active substances [5,6,7]. As such, they can be applied in drug delivery systems and the delivery of cosmetic components.

Notwithstanding these benefits, the volatility of ethanol gives rise to numerous limitations [8,9]. After an extended period of storing ethosomes, the ethanol component exhibits high volatility, resulting in the aggregation of ethosomes. This aggregation phenomenon can lead to an increase in particle size, the leakage of the encapsulated medication and the overall impaired stability of ethosomes. The restricted scope of its use significantly constrains its utility in the field of pharmaceutical research and development [10,11,12]. As such, binary ethosomes have been found as a promising carrier, and the synergistic effect of binary alcohol and ethanol can significantly increase the mobility and flexibility of the phospholipid bilayer, which leads to a significant difference compared to conventional ethosomes [13,14].

Propylene glycol (PG) is a colorless and viscous transparent liquid that has low volatility and induces little irritation. Due to the higher viscosity of PG, the structural stability of the lipid lamellar phase can be increased during the preparation of ethosomes. This addresses the limitations associated with the volatility of ethanol [15,16]. Manconi et al. incorporated thymus essential oil in glycerosomes and PG-containing vesicles that showed excellent stability during storage and maximized the antioxidant effect of the oil [17,18]. Wang demonstrated that two water-phase miscible osmotic promoters could be added to ethosomes to obtain a stable transdermal delivery system [19].

Previous studies have confirmed that ethosomes can effectively protect active substances, increase the solubility of hydrophobic substances and enhance bioavailability [20]. Ferrara et al. discovered that the application of ethosomes and transethosomes as carriers for quercetin can significantly increase the bioavailability and water solubility of quercetin, which greatly improves the entrapment capacity, antioxidant activity and stability of quercetin [21]. Kusumawati et al. found that compared with liposome, ethosomes comprise a more promising transdermal delivery carrier system, allowing Curcuma heyneana extract to penetrate deeper layers of the skin [22]. Phloretin (PHL), alternatively referred to as trihydroxy phenol acetone, is a flavonoid predominantly present in the peel and root bark of juicy fruits, such as apples and pears. It possesses a variety of physiological functions such as anti-inflammatory, anti-cancer and whitening properties [23,24]. Moreover, it exhibits robust antioxidant properties, allowing it to mitigate the detrimental effects of free radicals in the human body, thereby reducing oxidative stress-related cell damage. Due to its antioxidant properties, PHL has been demonstrated to mitigate the skin damage induced by UV radiation [25]. Nevertheless, the poor physicochemical stability and bioavailability of PHL greatly restricts its potential application in products [26,27]. As such, in this study, we explored the use of PG-stabilized vesicles in order to enhance the delivery of PHL and evaluated the antioxidant efficacy of PHL in ethosomes.

In this paper, PHL-loaded propylene glycol ethosomes (PHL-PGEs) were designed and developed to enhance the storage stability, skin permeability and antioxidative activity of PHL through encapsulation within binary ethosomal nanocarriers. This study used dynamic light scattering to determine particle size and zeta potential. Additionally, a set of stability tests were performed, encompassing assessments of salt tolerance, PH sensitivity, dilution multiples and storage stability. The measurement of molecular ordering, which is associated with the interaction between PHL and phospholipid membranes, was conducted using an X-ray diffractometer (XRD) and Fourier transform infrared spectroscopy (FTIR). A Franz diffusion cell was utilized to conduct in vitro infiltration experiments on mouse skin to compare the cumulative amount of skin infiltration. An assessment of antioxidative activity was ultimately conducted in order to validate the application potential of PGEs loaded with PHL. These ethosomes have potential for improving the carrying properties of drug carriers and in producing appropriate loading methods for future drug delivery studies.

## 2. Results and Discussion

### 2.1. Characterization of Ethosomes

**Figure 1:**
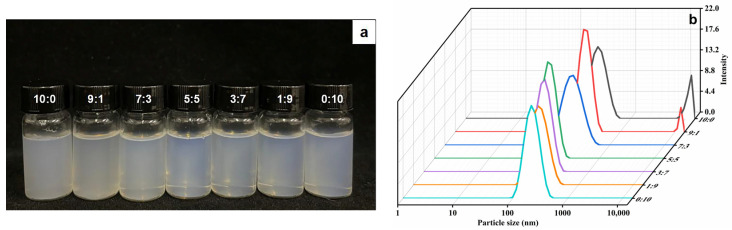
(**a**) Appearance pictures and (**b**) variation trend of particle size with EtOH/PG mass ratios of 10:0, 9:1, 7:3, 5:5, 3:7, 1:9 and 0:10.

The study effectively produced and optimized ethosomes that incorporated ethanol and PG with varying mass ratios. The aim of this study was to examine the variations among the aforementioned ethosomes according to their appearances, particle size and zeta potential. Firstly, the difference in the physical state of the sample is evident in Figure 1a. When no PG was added, the vesicle solution appeared to be white. As the proportion of PG increased, the vesicle solution exhibited enhanced transparency and displayed a characteristic blue emulsion phenomenon [28].

The particle size of PGEs ranged from 345.25 ± 8.25 nm (EtOH:PG = 10:0) to 212.54 ± 6.19 nm (EtOH:PG = 0:10), as indicated in Table 1. By gradually increasing the amount of PG, the particle size of PGEs was reduced to 119.27 nm when the EtOH/PG ratio was 5:5. This implies that there is an interaction between PG and the phospholipid bilayer, resulting in the increased flexibility of the bilayer and a reduction in particle size [29]. Similar trends were demonstrated in the results obtained for the PDI. Furthermore, it is evident that the EtOH/PG ratios of 10:0 and 9:1 exhibit a distinct bimodal distribution in Figure 1b. This observation suggests the presence of potentially significant aggregates inside the ethosome solution, which could potentially have impacted its stability. Consequently, more studies need to be conducted to assess the stability of the solution.

Vesicle surface charge, which is measured in terms of zeta potential, plays a crucial role in predicting both storage stability and the skin–vesicle interaction [30]. The zeta potential of all prepared PGEs had negative values ranging from −9.83 ± 0.67 to −17.4 ± 0.68 mV. This was increased by increasing the PG concentrations. The highest value was −25.7 ± 0.41 with a 5:5 EtOH/PG ratio. Systems had a high zeta potential due to the strong repulsion between charges, which reduced the cohesion between particles. This might be attributed to the fact that ethanol provides a concentration-dependent negative charge to polar heads of phospholipids that produces electrostatic repulsion and reduces vesicle aggregation, leading to durable stability [31].

### 2.2. Stability Study

#### 2.2.1. Salt Stability

**Figure 2:**
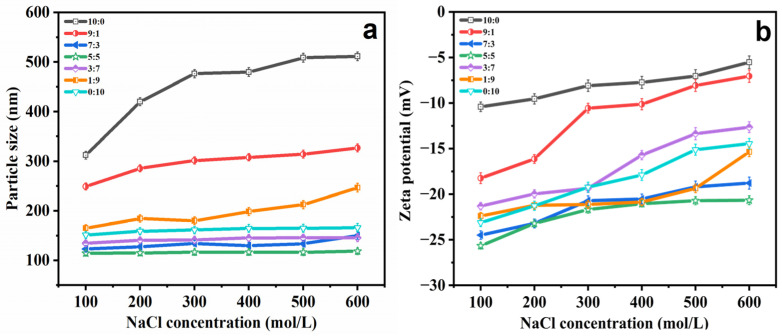
(**a**) Variations in particle size and (**b**) zeta potential of PGEs with EtOH/PG mass ratios of 10:0, 9:1, 7:3, 5:5, 3:7, 1:9 and 0:10 in NaCl solution with different concentrations (100−600 mM).

The presence of salt ions leads to poor stability and relatively high membrane semi-permeability in ethosomes, which is one of the main limitations in their use as a carrier [32]. Thus, the effect of different NaCl concentrations on ethosome suspension stability was examined by incubating them at room temperature for 12 h, as shown in Figure 2. With increasing concentrations of NaCl (0–600 mol/L), PGEs showed comparable stability when the ethanol:PG ratio was 7:3 to 1:9. The particle size falls within the range of 200 nm, whereas the potential ranges from −20 to −30 mV. In addition the particle size (Figure 2a) and zeta potential (Figure 2b) increased significantly in other ranges, which may have been due to the gradual dehydration of the ethosomes with the increased salt ion concentration, making the membrane more dense and rigid, and inducing less of a curvature, leading to the formation of larger ethosomes [33].

#### 2.2.2. pH Stability

**Figure 3:**
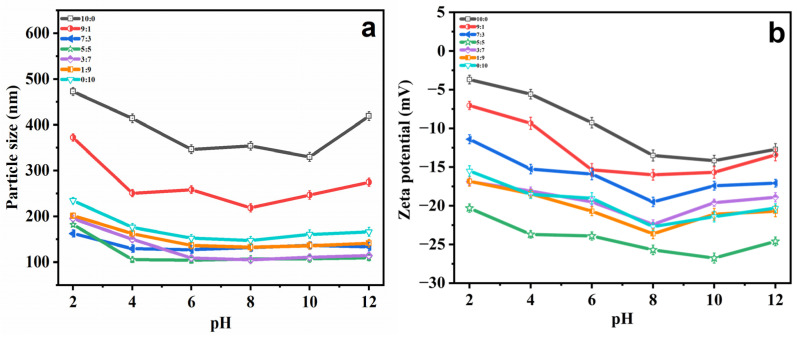
(**a**) Variations in particle size and (**b**) zeta potential of PGEs with EtOH/PG mass ratios of 10:0, 9:1, 7:3, 5:5, 3:7, 1:9 and 0:10 with different pHs (2, 4, 6, 8, 10 and 12).

Various pH conditions have impacts on the hydrolysis of ethosome phospholipid bilayer structures. Typically, ethosomes are prepared in the pH range of 4 to 10, and no significant hydrolysis reaction occurs for hours to days. However, in extreme pH conditions, hydrolysis occurs in a very short time period [34,35]. Therefore, various pH conditions (2.0, 4.0, 6.0, 8.0, 10.0 and 12.0) were selected to evaluate whether or not PG modification could increase the stability of ethosomes. In an environment with a pH of 4 to 8, the smallest vesicles (Figure 3a) and biggest zeta potential (Figure 3b) in size were observed. At other pHs, extreme acidity or alkalinity accelerated the hydrolysis process and destroyed the membrane structure, with this phenomenon being particularly noticeable without PG (EtOH:PG = 10:0) [36]. In conclusion, PG can enhance the pH stability of ethosomes.

#### 2.2.3. Dilution Multiple Stability

**Figure 4:**
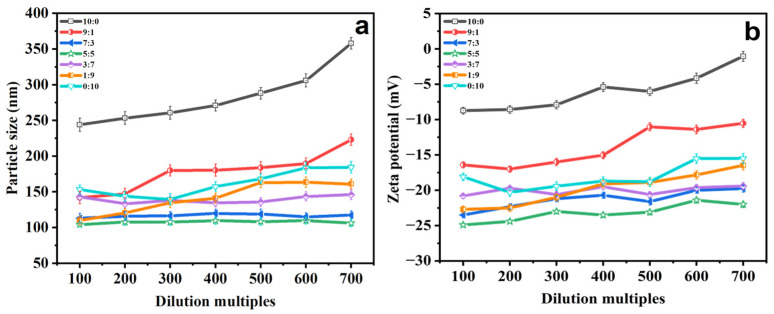
(**a**) Variations in particle size and (**b**) zeta potential of PGEs with EtOH/PG mass ratios of 10:0, 9:1, 7:3, 5:5, 3:7, 1:9 and 0:10 for different dilution multiples.

When PGEs are added to cosmetics, they generally need to be diluted before application. Hence, the particle size and zeta potential values of PGEs at different dilution multiples were determined to investigate the influence of different dilution multiples on the stability of the system [37,38]. As depicted in Figure 4a,b, the stability of the system experiences the most significant alteration in the absence of PG. The particle size exhibits an increase from 243.94 ± 9.32 to 358.04 ± 7.99 nm. The zeta potential decreases from −8.74 ± 0.48 to −1.05 ± 0.69 mV. This might be attributed to the slight aggregation of PGEs within this period [39]. Additionally the particle size and zeta potential of the other groups change slightly, demonstrating good dilution stability.

#### 2.2.4. Storage Stability

**Figure 5:**
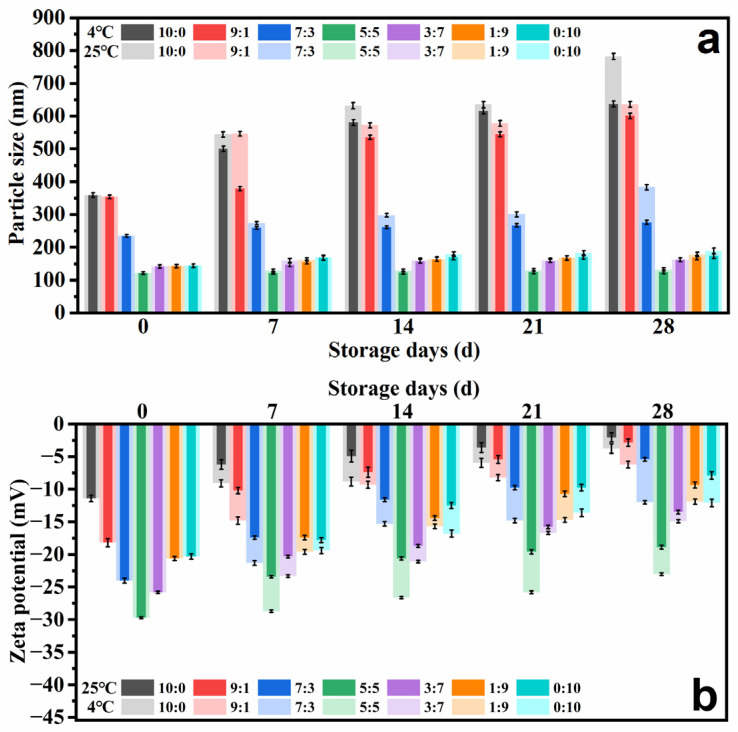
(**a**) Variations in particle size and (**b**) zeta potential of PGEs with EtOH/PG mass ratios of 10:0, 9:1, 7:3, 5:5, 3:7, 1:9 and 0:10 for 28 days of storage at 4 °C and 25 °C.

Aggregation between vesicles may occur during sample storage due to the influence of factors such as the environment [40]; thus, in order to examine storage stability, PGEs were prepared and stored for 28 days at 4 °C and 25 °C. The particle size and zeta potential of PGEs were determined, and the results are shown in Figure 5. Significant increases in particle size (Figure 5a) and zeta potential (Figure 5b) at 4 °C and 25 °C were observed for ratios of 10:1 and 9:1. This may indicate that the ethanol concentration was too large. A disruption of the amphiphilic nature of phospholipid molecules causes an almost complete dispersion of the membrane layer [41]. However, the particle size of the other PGEs remained stable during this storage period. It is worth noting that the particle size of PGEs stored at 25 °C was found to be higher than that of PGEs stored at 4 °C during the storage period. It is possible that the elevation in temperature enhances the fluidity of the PGEs membranes, leading to expedited growth in particle size [42]. These observations revealed that the presence of PG improves the stability of PGEs. In conclusion, the ethanol:PG ratio of 3:7 to 0:10 was selected as the optimal ratio for subsequent experiments.

### 2.3. Rheological Properties Analysis

**Figure 6:**
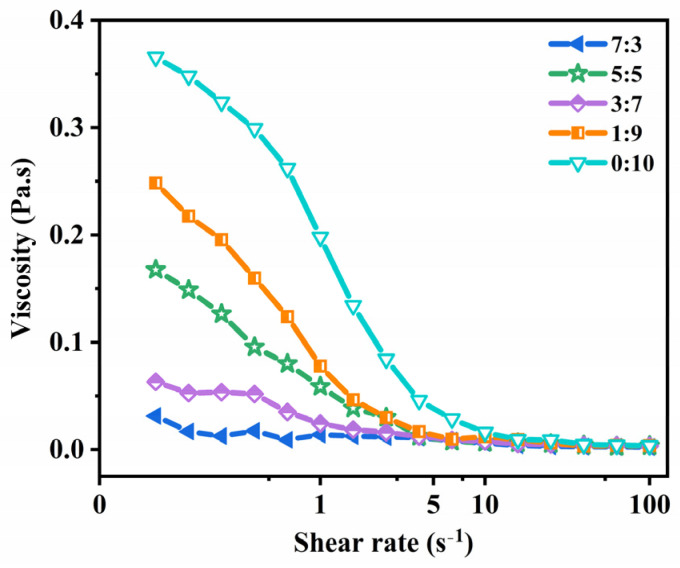
Trend chart of viscosity changes in ethosomes with EtOH/PG mass ratios of 7:3, 5:5, 3:7, 1:9 and 0:10.

The relationship between the shear rate and the apparent viscosity of the different ratios of ethanol and PG binary ethosomes (EtOH:PG = 7:3, 5:5, 3:7, 1:9 and 0:10) is shown in Figure 6. At high shear rates, every sample exhibited shear thinning behavior. Because the shear force disrupted the bridge between the emulsion droplets, the interactive forces maintaining stability were destroyed and shear thinning occurred [43,44]. Furthermore, the results displayed in Figure 6 reveal that EtOH/PG mass ratios of 0:10 and 1:9 have higher shear viscosity. This is caused by the viscosity of the samples, which increases with PG concentration. This results in the sample’s apparent viscosity rising, which promotes particle adherence [45,46]. Thus, during the ensuing studies, the EtOH/PG mass ratio was 5:5 based on the aforementioned parameters.

### 2.4. Encapsulation Efficiency and Drug Loading Capacity

The EE% and DL% (Table 2) of the optimized PGE formulations (referred to as PHL-PGEs) at different PHL concentrations were determined via the HPLC method. The results showed that the EE% ranged from 79.03 ± 2.13% to 89.42 ± 2.42%, while the DL% was between 3.16 ± 0.03% and 4.21 ± 0.04%. At a PHL concentration of 1 mg/mL, the EE% and DL% exhibited their highest values. As the concentration further increased, the values plateaued, suggesting that the double-layer film attained saturation in terms of PHL loading. Moreover, with the increase in PHL concentration, the particle size did not change significantly, and the dispersion was good. This implies that an elevation in PHL concentration will not result in the disruption of the double-layer membrane structure. Hence, following thorough deliberation and assessment, subsequent experiments were conducted at a PHL concentration of 1 mg/mL.

### 2.5. Transmission Electron Microscopy (TEM) Analysis

**Figure 7:**
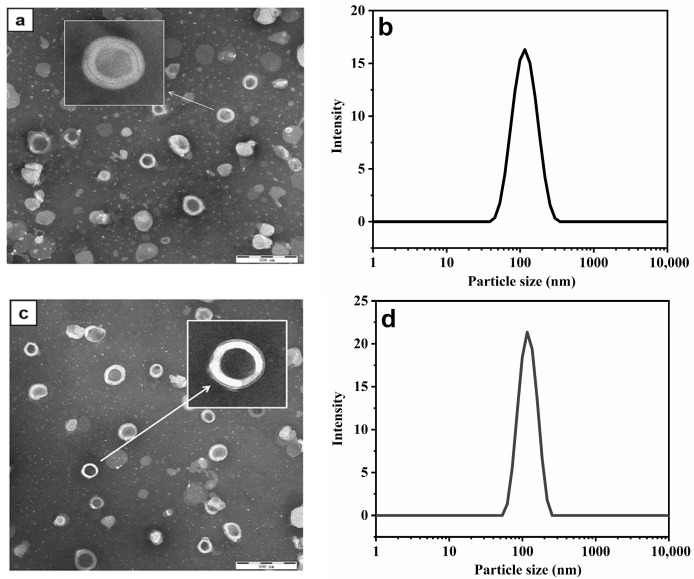
(**a**) TEM of PGEs; (**b**) particle size of PGEs; (**c**) TEM of PHL-PGEs; (**d**) particle size of PHL-PGEs.

Figure 7a,c display illustrations of the TEM of the PGEs and PHL-PGEs. Similar to previous lipid-based systems, they all exhibited uniform size, and the vesicles were devoid of noticeable aggregation [47]. Transmission electron microscopy at 100 nm showed a clearly visible double-layer structure and spherical appearance. Due to the loading of the drug, the particle size of PHL-PGEs is slightly larger than that of PGEs. However, there is no obvious difference in appearance [48]. The particle size corresponds to the dimensions shown in Figure 7b,d. The particle size of PGEs is around 117 nm, while the particle size of PHL-PGEs is around 122 nm. The smaller nano-size is closely related to skin permeability, as the small particle size allows for intimate interaction with the outer layer of the skin. This enhances the fluidity of the skin and facilitates the transportation of PHL to deeper layers [49].

### 2.6. X-ray Diffractometer (XRD) Analysis

**Figure 8:**
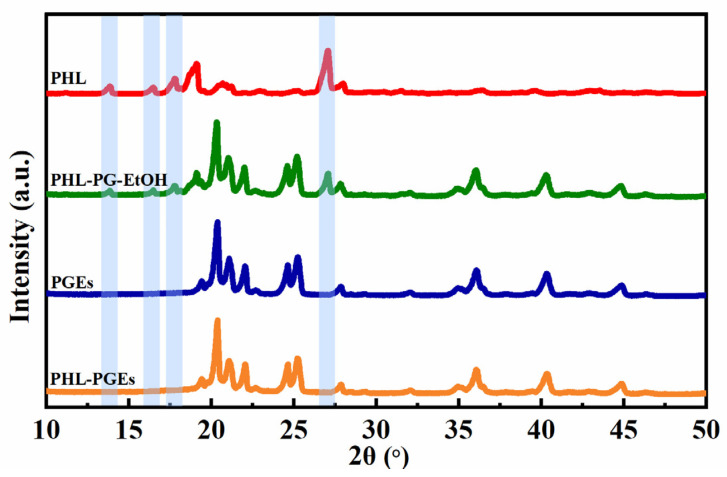
XRD spectrogram of PHL, PHL-PG-EtOH, PGEs and PHL-PGEs.

To enhance comprehension concerning the attributes of PHL-PGEs, an examination was carried out on the XRD profiles of pure PHL, PHL-PG-EtOH, PGEs and PHL-PGEs (Figure 8). The crystalline nature of PHL was confirmed by the presence of characteristic peaks found at 2θ values ranging from 10° to 50°, as shown in PHL. Furthermore, it can be observed in PHL-PG-EtOH that there is a distinct peak in the response corresponding to this particular position. This finding implies that the crystalline structure present in the mixture is the same as that of PHL. Nevertheless, it is evident that the spectrogram depicting PGEs exhibits an identical pattern to that in the spectrogram presented by PHL-PGEs, suggesting the absence of peaks associated with PHL in the spectra of PHL-PGLs. This observed phenomenon can potentially be ascribed to the formation of a shapeless compound of PHL, which arises from intermolecular interactions occurring within the matrix. Previous studies have recorded a similar phenomenon, providing evidence of the transformation of the crystalline structure into an amorphous state [50]. This demonstrates that the procedure of encapsulating PHL was effectively executed.

### 2.7. Fourier Transform Infrared (FTIR) Spectroscopy Analysis

**Figure 9:**
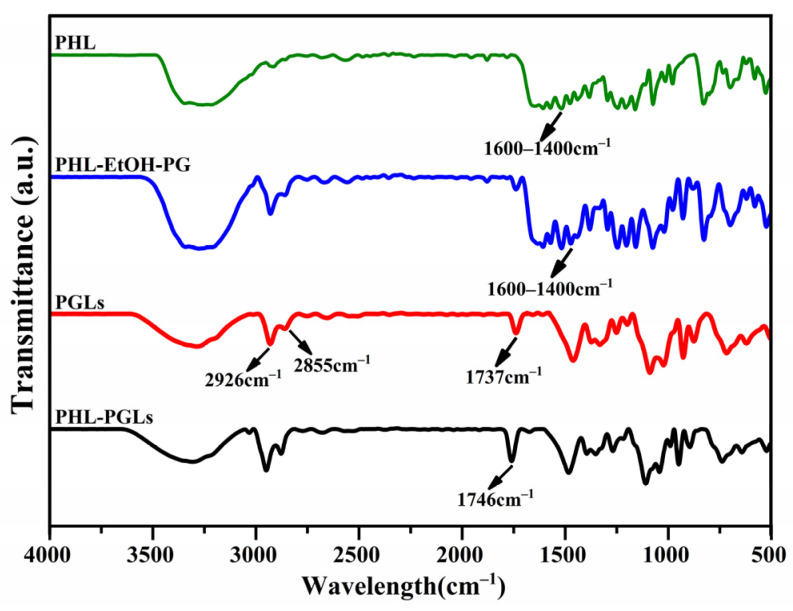
FTIR Spectrogram of PHL, PHL-PG-EtOH, PGEs and PHLPGEs.

As shown in Figure 9, vibration absorption peaks exhibiting PHL features were identified within the spectral range of 1600~1400 cm^−1^ [51]. The peak location of the PHL–PG–ETOH mixture remained unchanged, suggesting that the PHL and PGE components were only moderately mixed. Briefly, 2926 cm^−1^ and 2855 cm^−1^ are the vibrational absorption peaks of the hydrophobic group CH_2_ within the phospholipid bilayer. The stretching vibration at 1737 cm^−1^ corresponds to the presence of the (C=O) group in a lipid phospholipid. An alteration in the C=O peak signifies a modification in hydrogen bonding. After loading PHL, the C=O bond expansion vibration peak at 1737 cm^−1^ was blue-shifted to 1746 cm^−1^. This change implies that the PHL may be embedded in the phospholipid bilayers through hydrogen bonding and hydrophobic interaction, ultimately achieving successful encapsulation [52,53].

### 2.8. In Vitro Percutaneous Permeability Test

**Figure 10:**
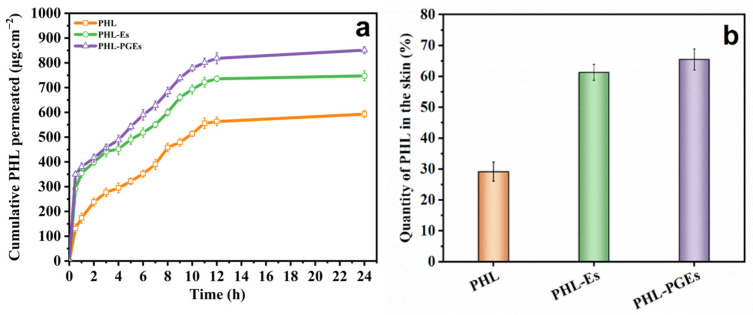
(**a**) Cumulative permeation and (**b**) drug retention of PHL from ethosomes and PGEs.

In order to investigate the enhancement effect of PHL-encapsulated binary ethosomes on permeation, percutaneous experiments were carried out with PHL ethanol solution, PHL-ethosomes (PHL-Es) and PHL-PGEs. Cumulative osmotic release curves of PHL across different formulations are graphed in Figure 10a, while skin retention is depicted in Figure 10b. The investigation involved evaluating the drug delivery capabilities of several systems by quantifying the drug quantity in the skin (Qm) as well as drug retention (Qn). The results reveal that skin permeability achieved by PHL-PGEs is superior to that achieved by the PHL-E and PHL solution. Ethosomes exhibit high permeability through the skin due to the existence of ethanol and propylene glycol (PG). This is due to the fact that the elevated concentrations of ethanol and PG contributes to the increased fluidity and flexibility of the binary ethosomes’ membranes. Consequently, this causes the deformation of the binary ethosomes during the transfer process and enhances their transdermal ability by inducing a disorder in the cuticle [24,54]. Nevertheless, when the concentration of ethanol is increased, it becomes more volatile, while propylene glycol (PG) aids in retaining the medication within the binary ethosomes. The sustained distribution of PHL-encapsulated ethosomes to the skin under closed conditions was achieved through the utilization of ethanol and PG throughout the preparation process. Additionally, the skin retention of PHL-PGEs was 1.06 times that of PHL-Es and 2.24 times that of PHL solution. These outcomes can be ascribed to the robust stability and substantial drug loadings of PHL-PGEs (EtOH:PG = 5:5). These properties enable the effective transportation of pharmaceuticals into the deeper layers of the skin, enhancing therapeutic efficacy. Consequently, the utilization of PHL-PGEs as drug carriers effectively prolongs the duration of the therapeutic benefits of PHL [30,55].

### 2.9. In Vitro Antioxidant Activity Study

**Figure 11:**
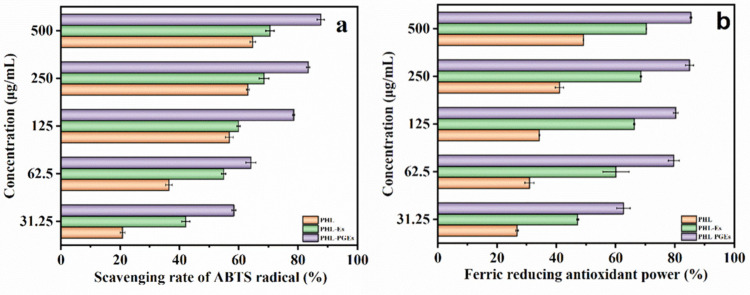

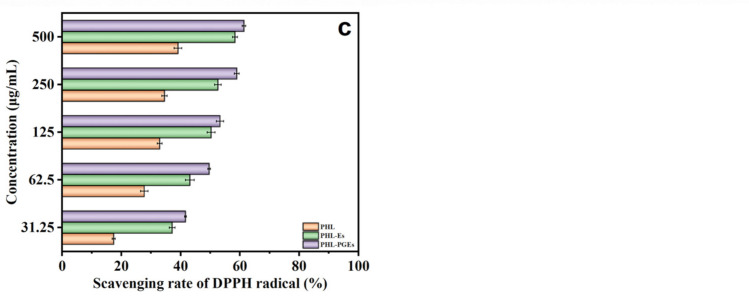
(**a**) ABTS radical scavenging activity, (**b**) FRAP and (**c**) DPPH radical scavenging activity of PHL, PHL-Es and PHL-PGEs.

The assessment of the antioxidant efficacy of PHL in ethosomes holds significance in guaranteeing that the formulation exhibits the intended degree of efficacy in safeguarding against oxidative harm [25]. Three distinct methodologies, namely DPPH, ABTS and Ferric reducing antioxidant power (FRAP), were used to assess the antioxidant activity of encapsulated PHL, with free PHL serving as the control. The ABTS free radical scavenging rate and FRAP of PHL were assessed in a manner that relied on the dose and type of PHL (Figure 11a,b). When the PHL concentration increased from 31.25 μg/mL to 500 μg/mL, the antioxidant activity gradually increased. At 31.25 μg/mL, the ABTS clearance rate and FRAP capacity of PHL-PGES were 55.32% and 62.65%, respectively, which were about 2.5 times higher than those of the PHL solution. The observed outcome can be ascribed to the presence of hydrophobic PHL, which was encased within nanoparticles possessing hydrophilic surfaces. This encapsulation facilitated the improved dispersion of PHL in water, hence enhancing the interaction between PHL and free radicals [56]. Ethosomes without the addition of PG have lower antioxidant potentials due to the presence of less carrier-coated PHL. In addition, it is worth noting that all samples demonstrated a relatively low level of DPPH radical clearance, as illustrated in Figure 11c. This level was consistently lower than that in the results obtained from the ABTS and FRAP tests, across all concentrations. The reason for this observation may be that the DPPH chromogenic solution dissolves in an organic solvent. Consequently, the vesicles are inclined to flocculate or cluster subsequent to their combination with DPPH reagents, thereby diminishing their efficacy in scavenging free radicals throughout the assay. This observation aligns with findings reported in prior studies [57]. In short, the utilization of PGEs for the nanoencapsulation of PHL is used to enhance its solubility in aqueous systems and safeguard it against degradation caused by UV light. This process facilitates the development of a durable nanostructure with antioxidant properties, thus presenting potential applications in topical treatments.

## 3. Materials and Methods

### 3.1. Materials

Soya lecthin, cholesterol, propylene glycol, sodium hydroxide and hydrochloric acid were acquired from Sinopharm Chemical Reagent Co., Ltd. (Shanghai, China). Anhydrous ethanol and methanol were supplied by Tianjin Kaitong Chemical Reagent Factory (Tianjin, China). PBS, phosphate-buffered solution, was supplied by Beijing Sora Biotechnology Co., Ltd. (Beijing, China). Potassium bromide was purchased from Shanghai Macklin Biochemical Co., Ltd. (99.5% of purity, Shanghai, China). Phloretin, propylene glycol, 1,1-Diphenyl-2-picryl hydrazyl radical (DPPH), 2,2′-azino-bis (3-ethylbenzothiazoline-6-sulfonic) (ABTS), salicylic acid, ferrous sulfate heptahydrate, hydrogen peroxide (30%) and potassium persulfate were purchased from Aladdin Biotechnology Co., Ltd. (Shanghai, China). All other reagents were of analytical grade, and all experiments used Milli-Q water.

### 3.2. Preparation of PGEs

PGEs were prepared in accordance with a previously described method [58]. The PGEs investigated were composed of phospholipid, cholesterol (CHO), ethanol solution (composed of EtOH and PG) and phosphate-buffered solution (PBS, pH = 6.8) (*v/v*). The phospholipid was dissolved in the ethanolic solution. PBS was gradually injected, while simultaneously mixing at a rotational speed of 700 rpm. The entire sample was continuously stirred for 30 min at 700 rpm in a closed thermostatic container. The system was maintained at 55 °C throughout the preparation procedure and then cooled to room temperature. The PGE suspension was passed through the membrane filter. Samples eluted during the initial 5 min were discarded. A series of PGEs were prepared. The weight ratios of ethanol to PG were 10:0, 9:1, 7:3, 5:5, 3:7, 1:9 and 0:10. For PHL-loaded PGEs (PHL-PGEs), phospholipid, CHO and PHL were dissolved with the phospholipid in an ethanol solution, and ethosomes without PG were used as a control group (PHL-Es).

### 3.3. Characterization of PGEs

The dynamic light scattering (DLS) technique was employed to measure particle size, polydispersity index (PDI) and zeta potential. This was accomplished using a Zetasizer Nano ZSE instrument (Malvern Instruments Ltd., Malvern, UK) equipped with a 633 nm He/Ne laser, with the detector positioned at a 90° angle. Particle size and the polydispersity index (PDI) are major constraints for the successful formulation of ethosomes as they can assist in the improvement of drug permeability and absorption [59]. The prepared ethosomes were diluted (10-fold) with Milli-Q water in order to mitigate the occurrence of multiple scattering. The zeta potential serves as a reliable measure of the potential stability of colloidal systems, wherein a higher magnitude of net zeta potential corresponds to the enhanced stability of nanoparticles [60]. Every sample was evaluated in triplicate at a temperature of 25 °C, with each measurement requiring 2 min pf equilibration prior to initiation.

### 3.4. Stability Study

#### 3.4.1. Salt Stability

Ethosomes were mixed with NaCl solutions in a volume ratio of 1:5. The concentration of NaCl in the mixed solution displayed fluctuation within a range of 100, 200, 300, 400, 500 and 600 mol/L. The particle size and zeta potential of the treated PGEs were assessed following a 12 h incubation period at ambient temperature.

#### 3.4.2. pH Stability

The pH of the ethosome suspension solution was changed by adding 0.1 mol/L of HCl or 0.1 mol/L sodium hydroxide, resulting in pH values of 2, 4, 6, 8, 10 and 12. The mixes were subjected to incubation at ambient temperature for a duration of 12 h. The study aimed to assess the impact of varying pH levels on the stability of the system by measuring changes in particle size and zeta potential.

#### 3.4.3. Dilution Multiples Stability

To examine the impact of various dilution multiples on the stability of the system, ethosome solutions were diluted 200, 300, 400, 500, 600, and 700 times using Milli-Q water and analyzed for particle size and zeta potential.

#### 3.4.4. Storage Stability

The ethosome samples were enclosed in glass vials, securely covered with Parafilm to prevent evaporation and subsequently stored at temperatures of 4 °C and 25 °C for durations of 0, 7, 14, 21 and 28 days. The samples were collected at the various time intervals and analyzed for particle size and zeta potential. The experiment involved the utilization of triplicate samples for testing purposes under various storage conditions [61].

### 3.5. Rheological Properties

The viscosity of the ethosome system was measured using a cone and plate combination with the aid of a rheometer (40 mm in diameter, with a 27 μm gap between the cone and plate, HR10, TA Instruments). The ethosome sample was positioned on the lower plate, and then, the upper plate was lowered to achieve the desired gap. The instrument software employed a conventional temperature stabilization technique to ensure that the sample attained and maintained the desired operational temperature. The oscillatory strain of the linear viscoelasticity was determined to be 1.5% by subjecting the linear region to oscillations at a temperature of 25 °C. The viscosity of the sample was assessed at ambient temperature and within the linear viscoelastic region by incrementally varying the shear rate from 0.1 to 100 s^−1^ to ensure the acquisition of reliable and consistent results [62].

### 3.6. High-Efficiency Liquid Chromatography (HPLC)

The measurement of the encapsulation efficiency (EE%) and drug loading capacity (DL%) of PHL was conducted using HPLC [63]. In this study, an Agilent C18 column (4.6 mm × 250 mm, 5 µm) was employed, with a mobile phase comprising a mixture of methanol and Milli-Q water. The flow rate of the mobile phase was established at 1.0 mL/min. The analyte was detected at a wavelength of 285 nm. Additionally, an injection volume of 10 µL was utilized for analysis.

### 3.7. Encapsulation Efficiency and Drug Loading Capacity

The primary parameters utilized for assessing drug encapsulation formation were EE% and DL% [64]. The PHL-PGEs were extracted precisely in volumes of 1 mL, thereafter diluted by a factor of 10 using methanol and subjected to sonication for a duration of 5 min in order to liberate PHL. The measurement was conducted using HPLC, and the concentration, C_1_, was determined. Following a process of accelerated centrifugation at a speed of 10,000 rpm for a duration of 1 h, the resulting supernatant was submitted to 10-fold dilution using methanol. Subsequently, the aforementioned solution was passed through a filter with a pore size of 0.22 μm. The filtrate was injected in accordance with the prescribed methodology, with a sample volume of 10 μL, and the concentration was obtained as C_2_. EE% was calculated using the following equation:

(1) $$EE\left(\%\right)=\frac{C_{2}}{C_{1}}$$

The freeze-dried powder of PHL-PGEs was denoted as W_2_. Thereafter, the powder was reconstituted with methanol, and the mass of PHL in the lyophilized powder was determined via HPLC and recorded as W_1_. DL% was calculated using the following equation:

(2) $$DL\left(\%\right)=\frac{W_{1}}{W_{2}}$$

### 3.8. Transmission Electron Microscopy (TEM)

The formulation was observed with the use of TEM (Hitachi H-750, HITACHI, Tokyo, Japan). The liposome sample was appropriately diluted using Milli-Q water. A slight amount of the diluted sample was then placed onto a copper grid and subsequently stained with 2% phosphotungstic acid solution. Following the drying process, each specimen was examined using a microscope with a magnification range of 10–100 k-fold, while maintaining an accelerating voltage within the range of 80–120 kV. A new grid was employed and subsequently observed for each sample [65].

### 3.9. X-ray Diffractometer (XRD)

The spectrograms of pure PHL, physical mixtures of PHL and PGEs (PHL-PG-EtOH), PGEs and PHL-PGEs were obtained using D8 Advance XRD (Bruker, Karlsruhe, Germany). The divergence slit was adjusted to an angle of 1°, while the reception slit was set to a width of 0.1 mm for the incident beam. The scanning rate employed in the study was 2°/min, ranging from 5° degrees to 50° at 2θ intervals [57].

### 3.10. Fourier Transform Infrared (FTIR) Spectroscopy

FTIR analysis was performed to check the chemical interaction between the excipients and the drug. The FTIR studies of PHL, PHL-PG-EtOH, PGEs and PHL-PGEs were achieved by using an FTIR instrument (Nicolet iS5 FTIR, Nicolet, Glendale, WI, USA) [66]. Prior to analysis, each sample was subjected to freeze-drying. Subsequently, the freeze-dried samples were individually combined with dried potassium bromide (KBr) in a 1:100 ratio. The resulting mixture was subsequently compressed into tablet form. In order to establish a baseline, it was necessary to measure a pure KBr tablet. The tablet was positioned within an infrared (IR) sample holder and subjected to scanning within the range of 4000–400 cm^−1^, employing a resolution of 4 cm^−1^, while maintaining room-temperature conditions.

### 3.11. In Vitro Percutaneous Permeability Test

Percutaneous experiments using Franz diffusion cells (Tianjin Pharmacopoeia Standard Instrument Factory, Tianjin, China) with an effective permeation area of 1.766 cm^2^ evaluated permeability (Qn) and skin retention (Qm) in PHL solutions, PHL-Es and PHL-PGEs [67]. The mouse skin diffusion membrane was positioned between the donor and receptor chambers, with the stratum corneum facing the donor chamber. To guarantee optimal contact between the mouse skin and the receiving fluid, a certain quantity of PBS (pH = 6.8) was introduced into the receptor chamber. Going through a 30 min period of equilibration, the samples, measuring 2 mL in volume, were administered onto the skin surface within the donor chamber. Subsequently, the chamber was securely sealed using parafilm. The temperature of the receptor medium was maintained at 37 ± 0.5 °C and it was continuously stirred at a speed of 200 rpm throughout the duration of the experiment using a receiving fluid. Briefly, 2 mL samples were obtained from the receiving fluid at various time intervals and promptly replaced with an equivalent volume of fresh receiving fluid at a temperature of 37 ± 0.5 °C. The analysis of the receiving fluid for drug content was conducted utilizing HPLC, as previously mentioned. Each investigation was conducted using triplicate experiments.

(3) $$Q_{n}=\frac{V_{0}\times C_{n}+\sum_{i=1}^{n-1}\left(C_{i}\times V_{i}\right)}{A}$$

The amount of PHL retained in the skin was determined following the completion of the in vitro permeation experiment (24 h). Mouse skin was rinsed with a Milli-Q-alcohol solution (50% *v/v* ethanol) to eliminate additional residue from the skin. The washed skin was subsequently cut into small pieces and subjected to homogenization using an alcohol-based solution in order to facilitate extraction. The solution obtained was subjected to centrifugation at a speed of 10,000 rpm for a duration of 5 min. The transparent liquid portion was gathered and subjected to HPLC for the purpose of drug content analysis following the aforementioned procedure. Each investigation was conducted in triplicate.

(4) $$Q_{m}\left(\%\right)=\frac{C_{m}\times A}{A}$$

### 3.12. Measurement of Antioxidative Activity

#### 3.12.1. DPPH Radical Scavenging Activity

The assessment of the antioxidant activity of PHL, as well as that of PHL-Es and PHL-PGEs, was conducted by examining their ability to scavenge DPPH radicals. The DPPH test involved the reduction of a violet-colored DPPH solution into a yellow-colored product, diphenylpicryl hydrazine, through the addition of an extract in a concentration-dependent manner. Various concentrations (31.25–500 µg/mL) of samples were prepared from the stock solution (1 mg/mL). The DPPH compound was dissolved in ethanol, resulting in the formation of a DPPH ethanol solution with a concentration of 0.20 mmol/L. Subsequently, a volume of 100 µL of DPPH solution was mixed with an equivalent volume of the samples. The solution was subjected to incubation for a duration of 30 min at 25 °C within an environment devoid of light. Absorbance (A_1_) was recorded at λ = 518 nm, using an enzyme-labeled instrument (Thermo Fisher Scientific Corporation, Vantaa, Finland). Milli-Q water mixed with the DPPH solution was used as a blank control (A_0_). The DPPH radical scavenging activity of the samples was determined by employing Equation (5):

(5) $$DPPH\;radical\;scavenging\;activity\left(\%\right)=\frac{A_{0}-A_{1}}{A_{0}}$$

#### 3.12.2. ABTS Radical Scavenging Activity

The determination of ABTS radical cation scavenging activity was conducted using a modified approach [68]. A solution of ABTS (7.4 mM) was combined with potassium persulfate (2.6 mM) and allowed to react for a duration of 12 h under dark conditions. Subsequently, the absorbance of the resulting combination was adjusted using ethanol to achieve a value of 0.70 ± 0.02 at a wavelength of 734 nm for the ABTS solution. Different concentrations of the sample (20 μL) were subjected to a reaction with ABTS (180 μL) for a duration of 10 min. Subsequently, the absorbance of the resulting mixture was measured at 732 nm and recorded as A_1_. The control substance was recorded as A_0_ using ethanol. The calculation of the antioxidant activity was performed using Equation (6):

(6) $$ABTS\;radical\;cation\;scavenging\;activity\left(\%\right)=\frac{A_{0}-A_{1}}{A_{0}}$$

#### 3.12.3. Measurement of Ferric Reducing Antioxidant Power

In terms of the Ferric reducing antioxidant power (FRAP) [69], 2.5 mL of the sample solution was blended with 2.5 mL of phosphate buffer (pH 6.6, 0.2 mol/L) and 2.5 mL of 1% (*w/v*) potassium ferricyanide solution. Following a 20 min incubation period at a temperature of 50 °C, a volume of 2.5 mL of trichloroacetic acid (10%) was added into the solution. Subsequently, the resulting mixture underwent centrifugation at a speed of 5000 rpm for a duration of 10 min. Next, a volume of 2.5 mL of the supernatant was combined with 2.5 mL of Milli-Q water and 0.5 mL of ferric chloride solution at a concentration of 0.1%. Then, the samples were placed in a period of darkness lasting 10 min, after which the absorbance (A_1_) of the samples was quantified at 700 nm. The absorbance (A_0_) used measured Milli-Q water as control. FRAP was calculated according to the following equation:

(7) $$FRAP\left(\%\right)=\left(A_{1}-A_{0}\right)$$

## 4. Conclusions

In this study, PHL-loaded binary ethosomes with different EtOH/PG mass ratios were successfully prepared. The augmentation of PG content reduced dimensions of vesicles, enhancing the effectiveness of encapsulation and the stability of the ethosomes. The in vitro percutaneous permeability test indicated that EtOH and PG can significantly enhance its skin permeability, and that the permeability effect of PHL-PGEs is far better than that of PHL-Es. The in vitro antioxidant activity study indicated that the antioxidant activity was potentiated by the co-nanoencapsulation of PHL. Further research on the suitable animal models is needed to evaluate the potential of the binary ethosomes as cosmetic ingredients.

## Figures and Tables

**Table 1:** Particle size, PDI and zeta potential of empty vesicles for different proportions of EtOH and PG (EtOH:PG = 0:10, 9:1, 7:3, 5:5, 3:7, 1:9 and 0:10).

Ethosomes Formulations	Particle Size (nm)	PDI	Zeta Potential (mV)
10:0	345.25 ± 8.25	0.443 ± 0.008	−9.83 ± 0.67
9:1	273.32 ± 6.43	0.318 ± 0.009	−15.8 ± 0.58
7:3	193.78 ± 4.74	0.201 ± 0.004	−23.4 ± 0.32
5:5	119.27 ± 4.50	0.161 ± 0.002	−25.7 ± 0.41
3:7	153.26 ± 5.67	0.064 ± 0.003	−22.3 ± 0.52
1:9	184.98 ± 6.31	0.072 ± 0.005	−18.4 ± 0.49
0:10	212.54 ± 6.19	0.075 ± 0.004	−17.4 ± 0.68

**Table 2:** Particle size, PDI, EE% and DL% of PHL-PGEs at different PHL concentrations.

PHL Concentration (mg/mL)	Particle Size (nm)	PDI	EE%	DL%
0.50	114.87 ± 4.65	0.125 ± 0.003	79.03 ± 2.13	3.16 ± 0.03
0.75	117.00 ± 3.75	0.129 ± 0.002	82.37 ± 1.94	3.75 ± 0.02
1.00	122.94 ± 4.34	0.154 ± 0.004	89.42 ± 2.42	4.21 ± 0.04
1.25	129.73 ± 3.98	0.158 ± 0.007	89.12 ± 2.17	4.16 ± 0.05
1.5	127.87 ± 4.12	0.156 ± 0.006	89.23 ± 2.25	4.19 ± 0.04

## Data Availability

The data presented in this study are available on request from the corresponding author. The data are not publicly available due to privacy.

## References

[1] Apolinário A.C., Salata G.C., de Souza M.M., Chorilli M., Lopes L.B. (2022). Rethinking Breast Cancer Chemoprevention: Technological Advantages and Enhanced Performance of a Nanoethosomal-Based Hydrogel for Topical Administration of Fenretinide. AAPS PharmSciTech.

[2] Touitou E., Dayan N., Bergelson L., Godin B., Eliaz M. (2000). Ethosomes—Novel Vesicular Carriers for Enhanced Delivery: Characterization and Skin Penetration Properties. J. Control. Release.

[3] Elizabeth S. (1983). Rowe Lipid Chain Length and Temperature Dependence of Ethanol-Phosphatidylcholine Interactions. Biochemistry.

[4] Adnan M., Akhter M.H., Afzal O., Altamimi A.S.A., Ahmad I., Alossaimi M.A., Jaremko M., Emwas A.-H., Haider T., Haider M.F. (2023). Exploring Nanocarriers as Treatment Modalities for Skin Cancer. Molecules.

[5] Limongi T., Susa F., Marini M., Allione M., Torre B., Pisano R., Di Fabrizio E. (2021). Lipid-Based Nanovesicular Drug Delivery Systems. Nanomaterials.

[6] Lu J., Guo T., Fan Y., Li Z., He Z., Yin S., Feng N. (2021). Recent Developments in the Principles, Modification and Application Prospects of Functionalized Ethosomes for Topical Delivery. Curr. Drug Deliv..

[7] Zhigaltsev I.V., Maurer N., Akhong Q.F., Leone R., Leng E., Wang J., Semple S.C., Cullis P.R. (2005). Liposome-Encapsulated Vincristine, Vinblastine and Vinorelbine: A Comparative Study of Drug Loading and Retention. J. Control. Release.

[8] Ruan S., Zhang Y., Feng N. (2021). Microneedle-Mediated Transdermal Nanodelivery Systems: A Review. Biomater. Sci..

[9] Paiva-Santos A.C., Silva A.L., Guerra C., Peixoto D., Pereira-Silva M., Zeinali M., Mascarenhas-Melo F., Castro R., Veiga F. (2021). Ethosomes as Nanocarriers for the Development of Skin Delivery Formulations. Pharm. Res..

[10] Sguizzato M., Ferrara F., Hallan S.S., Baldisserotto A., Drechsler M., Malatesta M., Costanzo M., Cortesi R., Puglia C., Valacchi G. (2021). Ethosomes and Transethosomes for Mangiferin Transdermal Delivery. Antioxidants.

[11] Ma H., Guo D., Fan Y., Wang J., Cheng J., Zhang X. (2018). Paeonol-Loaded Ethosomes as Transdermal Delivery Carriers: Design, Preparation and Evaluation. Molecules.

[12] Soleymani J., Jouyban-Gharamaleki V., Suleymanov T.A., Jouyban-Gharamaleki K., Jouyban A. (2017). Solubilization of Lamotrigine Using Tween 80 and Ethylene Glycol or Propylene Glycol. J. Mol. Liq..

[13] Elsayed M.M.A., Abdallah O.Y., Naggar V.F., Khalafallah N.M. (2007). Lipid Vesicles for Skin Delivery of Drugs: Reviewing Three Decades of Research. Int. J. Pharm..

[14] Manconi M., Mura S., Sinico C., Fadda A.M., Vila A.O., Molina F. (2009). Development and Characterization of Liposomes Containing Glycols as Carriers for Diclofenac. Colloids Surfaces A Physicochem. Eng. Asp..

[15] Aljohani A.A., Alanazi M.A., Munahhi L.A., Hamroon J.D., Mortagi Y., Qushawy M., Soliman G.M. (2023). Binary Ethosomes for the Enhanced Topical Delivery and Antifungal Efficacy of Ketoconazole. OpenNano.

[16] Li W.-Z., Hao X.-L., Zhao N., Han W.-X., Zhai X.-F., Zhao Q., Wang Y.-E., Zhou Y.-Q., Cheng Y.-C., Yue Y.-H. (2016). Propylene Glycol-Embodying Deformable Liposomes as a Novel Drug Delivery Carrier for Vaginal Fibrauretine Delivery Applications. J. Control. Release.

[17] Manconi M., Petretto G., D’hallewin G., Escribano E., Milia E., Pinna R., Palmieri A., Firoznezhad M., Peris J.E., Usach I. (2018). Thymus Essential Oil Extraction, Characterization and Incorporation in Phospholipid Vesicles for the Antioxidant/Antibacterial Treatment of Oral Cavity Diseases. Colloids Surfaces B Biointerfaces.

[18] Dickey A.N., Faller R. (2007). How Alcohol Chain-Length and Concentration Modulate Hydrogen Bond Formation in a Lipid Bilayer. Biophys. J..

[19] Wang H., Shao Q., Zhang Y., Ding J., Yang M., Yang L., Wang W., Cui P., Dai Z., Ma L. (2023). Preparation and Evaluation of Liposomes Containing Ethanol and Propylene Glycol as Carriers for Nicotine. Curr. Drug Deliv..

[20] Akhtar N., Akhtar N. (2022). Development of Stable Tocopherol Succinate-loaded Ethosomes to Enhance Transdermal Permeation: In Vitro and in Vivo Characterizations. J. Cosmet. Dermatol..

[21] Ferrara F., Benedusi M., Sguizzato M., Cortesi R., Baldisserotto A., Buzzi R., Valacchi G., Esposito E. (2022). Ethosomes and Transethosomes as Cutaneous Delivery Systems for Quercetin: A Preliminary Study on Melanoma Cells. Pharmaceutics.

[22] Kusumawati I., Kurniawan K.O., Rohmania R., Pratama B.A., Pratama Y.A., Rullyansyah S., Warsito M.F., Widyowati R., Hestianah E.P., Matsunami K. (2023). Comparative Study of Liposomal and Ethosomal Formulations of Curcuma Heyneana Rhizome Extract in a Transdermal Delivery System. Pharm. Nanotechnol..

[23] Anunciato Casarini T.P., Frank L.A., Pohlmann A.R., Guterres S.S. (2020). Dermatological Applications of the Flavonoid Phloretin. Eur. J. Pharmacol..

[24] Pawlikowska-Pawlega B., Ignacy Gruszecki W., Misiak L., Paduch R., Piersiak T., Zarzyka B., Pawelec J., Gawron A. (2007). Modification of Membranes by Quercetin, a Naturally Occurring Flavonoid, via Its Incorporation in the Polar Head Group. Biochim. Biophys. Acta Biomembr..

[25] Deshpande R.D., Shah D.S., Gurram S., Jha D.K., Batabyal P., Amin P.D., Sathaye S. (2023). Formulation, Characterization, Pharmacokinetics and Antioxidant Activity of Phloretin Oral Granules. Int. J. Pharm..

[26] Shin S., Kum H., Ryu D., Kim M., Jung E., Park D. (2014). Protective Effects of a New Phloretin Derivative against UVB-Induced Damage in Skin Cell Model and Human Volunteers. Int. J. Mol. Sci..

[27] Luo F.-C., Zhu J.-J., You X.-M., Yang X.-Q., Yin S.-W. (2023). Biocompatible Gliadin-Sericin Complex Colloidal Particles Used for Topical Delivery of the Antioxidant Phloretin. Colloids Surfaces B Biointerfaces.

[28] Li J., Chang C., Chen W., Su Y., Gu L., Yang Y., Zhai J. (2022). Hybrid Liposomes Composed of Hydrophilic Emulsifiers and Lecithin: Physicochemical, Interaction and Curcumin Loading Properties. Colloids Surfaces A Physicochem. Eng. Asp..

[29] Elmoslemany R.M., Abdallah O.Y., El-Khordagui L.K., Khalafallah N.M. (2012). Propylene Glycol Liposomes as a Topical Delivery System for Miconazole Nitrate: Comparison with Conventional Liposomes. AAPS PharmSciTech.

[30] Jafari A., Daneshamouz S., Ghasemiyeh P., Mohammadi-Samani S. (2023). Ethosomes as Dermal/Transdermal Drug Delivery Systems: Applications, Preparation and Characterization. J. Liposome Res..

[31] Hajare A.A., Dol H.S. (2021). Screening of Effective Formulation Techniques for Designing and Fabrication of Terbinafine Hydrochloride Ethosomes. Res. J. Pharm. Technol..

[32] Phatale V., Vaiphei K.K., Jha S., Patil D., Agrawal M., Alexander A. (2022). Overcoming Skin Barriers through Advanced Transdermal Drug Delivery Approaches. J. Control. Release.

[33] Mouritsen O.G. (2011). Lipids, Curvature, and Nano-medicine. Eur. J. Lipid Sci. Technol..

[34] Xu D., Xie J., Feng X., Zhang X., Ren Z., Zheng Y., Yang J. (2020). Preparation and Evaluation of a Rubropunctatin-Loaded Liposome Anticancer Drug Carrier. RSC Adv..

[35] Hassane Hamadou A., Zhang J., Chao C., Xu B. (2022). Stability of Rutin Using Pectin-Chitosan Dual Coating Nanoliposomes. LWT.

[36] Wang X., Swing C.J., Feng T., Xia S., Yu J., Zhang X. (2020). Effects of Environmental PH and Ionic Strength on the Physical Stability of Cinnamaldehyde-Loaded Liposomes. J. Dispers. Sci. Technol..

[37] Zhang T., Zhou S., Liu Y., Luo X., Di D., Song Y., Liu X., Deng Y. (2017). Polysialic Acid and Pluronic F127 Mixed Polymeric Micelles of Docetaxel as New Approach for Enhanced Antitumor Efficacy. Drug Dev. Ind. Pharm..

[38] Huang S., Wang X., Liu M., Lin Z., Gu W., Zhao H., Zhang Y., Ding B., Liu J., Wu X. (2022). Modification of Sodium Aescinate into a Safer, More Stable and Effective Water-Soluble Drug by Liposome-Encapsulation: An in Vitro and in Vivo Study. Drug Deliv..

[39] Sakdiset P., Amnuaikit T., Pichayakorn W., Pinsuwan S. (2019). Formulation Development of Ethosomes Containing Indomethacin for Transdermal Delivery. J. Drug Deliv. Sci. Technol..

[40] Junyaprasert V.B., Singhsa P., Suksiriworapong J., Chantasart D. (2012). Physicochemical Properties and Skin Permeation of Span 60/Tween 60 Niosomes of Ellagic Acid. Int. J. Pharm..

[41] Ghiasi F., Eskandari M.H., Golmakani M.T., Rubio R.G., Ortega F. (2021). Build-Up of a 3D Organogel Network within the Bilayer Shell of Nanoliposomes. A Novel Delivery System for Vitamin D3: Preparation, Characterization, and Physicochemical Stability. J. Agric. Food Chem..

[42] Pilch E., Musiał W. (2018). Liposomes with an Ethanol Fraction as an Application for Drug Delivery. Int. J. Mol. Sci..

[43] Vecher O.V., Diskaeva E.I., Bazikov I.A., Elbekyan K.S., Diskaeva E.N. (2020). Study of Some Rheological Properties of Niosomal Dispersions of Various Concentrations Based on PEG-12 Dimethicone. Adv. Nat. Sci. Nanosci. Nanotechnol..

[44] Hasan M., Ben Messaoud G., Michaux F., Tamayol A., Kahn C.J.F., Belhaj N., Linder M., Arab-Tehrany E. (2016). Chitosan-Coated Liposomes Encapsulating Curcumin: Study of Lipid-Polysaccharide Interactions and Nanovesicle Behavior. RSC Adv..

[45] Ali S., Davinelli S., Mencucci R., Fusi F., Scuderi G., Costagliola C., Scapagnini G. (2021). Crosslinked Hyaluronic Acid with Liposomes and Crocin Confers Cytoprotection in an Experimental Model of Dry Eye. Molecules.

[46] Sun Y., Tang W., Pu C., Li R., Sun Q., Wang H. (2022). Improved Stability of Liposome-Stabilized Emulsions as a Co-Encapsulation Delivery System for Vitamin B2, Vitamin E and β-Carotene. Food Funct..

[47] Manconi M., Caddeo C., Nacher A., Diez-Sales O., Peris J.E., Ferrer E.E., Fadda A.M., Manca M.L. (2019). Eco-Scalable Baicalin Loaded Vesicles Developed by Combining Phospholipid with Ethanol, Glycerol, and Propylene Glycol to Enhance Skin Permeation and Protection. Colloids Surfaces B Biointerfaces.

[48] Jiang J., Ma T., Zhang L., Cheng X., Wang C. (2020). The Transdermal Performance, Pharmacokinetics, and Anti-Inflammatory Pharmacodynamics Evaluation of Harmine-Loaded Ethosomes. Drug Dev. Ind. Pharm..

[49] Arunprasert K., Pornpitchanarong C., Piemvuthi C., Siraprapapornsakul S., Sripeangchan S., Lertsrimongkol O., Opanasopit P., Patrojanasophon P. (2022). Nanostructured Lipid Carrier-Embedded Polyacrylic Acid Transdermal Patches for Improved Transdermal Delivery of Capsaicin. Eur. J. Pharm. Sci..

[50] Song F., Yang G., Wang Y., Tian S. (2022). Effect of Phospholipids on Membrane Characteristics and Storage Stability of Liposomes. Innov. Food Sci. Emerg. Technol..

[51] Li X.M., Zhu J., Pan Y., Meng R., Zhang B., Chen H.Q. (2019). Fabrication and Characterization of Pickering Emulsions Stabilized by Octenyl Succinic Anhydride -Modified Gliadin Nanoparticle. Food Hydrocoll..

[52] Ding L., Yang J., Yin K., Cheng H., Li J., Xue C. (2022). The Spatial Arrangement of Astaxanthin in Bilayers Greatly Influenced the Structural Stability of DPPC Liposomes. Colloids Surfaces B Biointerfaces.

[53] Forutan M., Hasani M., Hasani S., Salehi N., Sabbagh F. (2022). Liposome System for Encapsulation of Spirulina Platensis Protein Hydrolysates: Controlled-Release in Simulated Gastrointestinal Conditions, Structural and Functional Properties. Materials.

[54] Ho M.J., Park H.J., Kang M.J. (2023). Neutral Oil-Incorporated Liposomal Nanocarrier for Increased Skin Delivery of Ascorbic Acid. Materials.

[55] Nainwal N., Jawla S., Singh R., Saharan V.A. (2019). Transdermal Applications of Ethosomes—A Detailed Review. J. Liposome Res..

[56] Cassano R., Curcio F., Sole R., Trombino S. (2023). Transdermal Delivery of Phloretin by Gallic Acid Microparticles. Gels.

[57] Wang J., Zhao Y., Zhai B., Cheng J., Sun J., Zhang X., Guo D. (2023). Phloretin Transfersomes for Transdermal Delivery: Design, Optimization, and In Vivo Evaluation. Molecules.

[58] Moolakkadath T., Aqil M., Ahad A., Imam S.S., Praveen A., Sultana Y., Mujeeb M., Iqbal Z. (2019). Fisetin Loaded Binary Ethosomes for Management of Skin Cancer by Dermal Application on UV Exposed Mice. Int. J. Pharm..

[59] Abouhussein D.M.N. (2021). Enhanced Transdermal Permeation of BCS Class IV Aprepitant Using Binary Ethosome: Optimization, Characterization and Ex Vivo Permeation. J. Drug Deliv. Sci. Technol..

[60] Kubiliene A., Munius E., Songailaite G., Kokyte I., Baranauskaite J., Liekis A., Sadauskiene I. (2023). A Comparative Evaluation of Antioxidant Activity of Extract and Essential Oil of *Origanum Onites* L. In Vivo. Molecules.

[61] Saewan N., Jimtaisong A., Panyachariwat N., Chaiwut P. (2023). In Vitro and In Vivo Anti-Aging Effect of Coffee Berry Nanoliposomes. Molecules.

[62] Abdelkader H., Wu Z., Al-Kassas R., Alany R.G. (2012). Niosomes and Discomes for Ocular Delivery of Naltrexone Hydrochloride: Morphological, Rheological, Spreading Properties and Photo-Protective Effects. Int. J. Pharm..

[63] Xiang Q., Wang M., Chen F., Gong T., Jian Y., Zhang Z., Huang Y. (2007). Lung-Targeting Delivery of Dexamethasone Acetate Loaded Solid Lipid Nanoparticles. Arch. Pharm. Res..

[64] Chen J., Fang W., Liu W., Liu J., Gong P. (2023). Microcapsules and Nanoliposomes Based Strategies to Improve the Stability of Blueberry Anthocyanins. Molecules.

[65] Palchoudhury S., Das P., Ghasemi A., Tareq S.M., Sengupta S., Han J., Maglosky S., Almanea F., Jones M., Cox C. (2023). A Novel Experimental Approach to Understand the Transport of Nanodrugs. Materials.

[66] Tiţa B., Fuliaş A., Bandur G., Marian E., Tiţa D. (2011). Compatibility Study between Ketoprofen and Pharmaceutical Excipients Used in Solid Dosage Forms. J. Pharm. Biomed. Anal..

[67] Kumar B., Sahoo P.K. (2023). Augmented Transdermal Delivery of Curcumin for the Effective Management of Plaque Psoriasis—Design, Formulation, Characterisation, and In Vivo Studies. AAPS PharmSciTech.

[68] Jo Y.J., Cho H.S., Chun J.Y. (2021). Antioxidant Activity of β-Cyclodextrin Inclusion Complexes Containing Trans-Cinnamaldehyde by DPPH, ABTS and FRAP. Food Sci. Biotechnol..

[69] Müller L., Fröhlich K., Böhm V. (2011). Comparative Antioxidant Activities of Carotenoids Measured by Ferric Reducing Antioxidant Power (FRAP), ABTS Bleaching Assay (ATEAC), DPPH Assay and Peroxyl Radical Scavenging Assay. Food Chem..

